# Graphene-Based Sensing Platform for On-Chip Ochratoxin A Detection

**DOI:** 10.3390/toxins11100550

**Published:** 2019-09-20

**Authors:** Nikita Nekrasov, Dmitry Kireev, Aleksei Emelianov, Ivan Bobrinetskiy

**Affiliations:** 1 National Research University of Electronic Technology, Zelenograd, 124498 Moscow, Russia; 8141147@gmail.com (N.N.); emmsowton@gmail.com (A.E.); 2 Department of Electrical and Computer Engineering, The University of Texas at Austin, Austin, TX 78712, USA; d.kireev@utexas.edu; 3 P.N. Lebedev Physical Institute of the Russian Academy of Sciences, 119991 Moscow, Russia; 4 BioSense Institute—Research and Development Institute for Information Technologies in Biosystems, University of Novi Sad, 21000 Novi Sad, Serbia

**Keywords:** graphene, transistor, ochratoxin A, aptamer, sensor, on-chip

## Abstract

In this work, we report an on-chip aptasensor for ochratoxin A (OTA) toxin detection that is based on a graphene field-effect transistor (GFET). Graphene-based devices are fabricated via large-scale technology, allowing for upscaling the sensor fabrication and lowering the device cost. The sensor assembly was performed through covalent bonding of graphene’s surface with an aptamer specifically sensitive towards OTA. The results demonstrate fast (within 5 min) response to OTA exposure with a linear range of detection between 4 ng/mL and 10 pg/mL, with a detection limit of 4 pg/mL. The regeneration time constant of the sensor was found to be rather small, only 5.6 s, meaning fast sensor regeneration for multiple usages. The high reproducibility of the sensing response was demonstrated via using several recycling procedures as well as various GFETs. The applicability of the aptasensor to real samples was demonstrated for spiked red wine samples with recovery of about 105% for a 100 pM OTA concentration; the selectivity of the sensor was also confirmed via addition of another toxin, zearalenone. The developed platform opens the way for multiplex sensing of different toxins using an on-chip array of graphene sensors.

## 1. Introduction

Mycotoxins—food contaminants that are sequestered at different steps of production, like planting, harvesting, processing, and storage—can cause multiple foodborne diseases in animals and humans, including liver and kidney disease and impaired growth [1,2]. Many countries are dealing with this problem by establishing stringent legislation surrounding food control. Nevertheless, due to the rising volume of food consumption and the increased complexity of food production chains, nowadays there is a considerable risk of delivering mycotoxins even down to households. Thus, there is a need for the implementation of simple, fast, non-expensive, selective, and yet accurate food quality control methods, preferably integrated into a portable sensor system or a smartwatch concept. Such sensing technologies and devices are typically based on a combination of micro- and nano-fabricated materials and large-scale CMOS (complementary metal-oxide-semiconductor) technology has huge potential to be used for personal food safety control [3].

Ochratoxin A (OTA) is one of the most widespread foodborne toxins [2,4]. It can be found in different products like cereals, meat, fruits, wine, beer, coffee, etc., and at different steps of food production. The toxin affects mostly the kidney, leading to a chronic disease called Balkan endemic nephropathy [5]. Like most of the mycotoxins, OTA cannot be destroyed during thermal treatment and is stable during fermentation. The only way to prevent exposure to OTA in animals and humans is via quick and precise detection prior to food utilization.

Different countries have strict regulations for the maximum tolerated level of OTA, which varies for different foods and is in the order of several parts per billion (ppb) [6]. Currently, chromatography is used for the detection of OTA concentrations below this level, yet the measurements must be done with proper sampling methods [6]. Although chromatography is highly accurate, it is slow and very complicated in both operation and results analysis. Another way is based on small portable sensors such as a lateral flow strip [7], surface plasmon resonance [8], or electrochemical methods [9,10], including screen-printed electrodes [11]. These methods provide fast and easy-to-operate onsite diagnostics but with accuracy that still must be improved.

In recent years, aptasensors based on graphene transistors have become powerful as tools addressing both issues of measurement speed and detection accuracy for different analytes, especially in the food industry [12]. Due to the excellent electrical properties of graphene and robust electrolyte–gate coupling, graphene field-effect transistors (GFETs) covalently functionalized with aptamers have demonstrated an unprecedented sensitivity to single-stranded DNA [13], mercury [14], insulin [15], immunoglobulin [16], ATP [17], and many others. Nonetheless, the development of aptamer-modified GFETs for mycotoxin detection has not been demonstrated until now.

**Figure 1:**
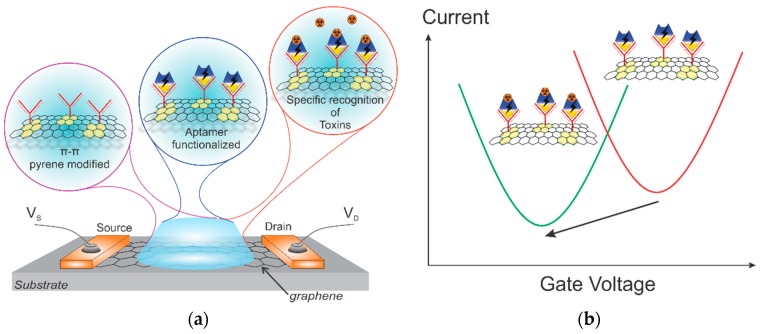
Scheme (**a**) and principle (**b**) of the graphene field-effect transistor (GFET) aptasensor for toxin detection

This work reports on the fabrication of a rapid and ultrasensitive sensor assay that is based on a GFET covalently bonded to a specific aptamer for the detection of ochratoxin A. A schematic of the device and its operation principle are shown in Figure 1. While graphene serves as a channel to the transistor, the aptamer is covalently grafted to the graphene’s surface via an intermediate ester and π–π interaction (see Figure 1a). The toxins later react with the OTA aptamer, resulting in a further shift of graphene’s transfer I–V curve (see Figure 1b). Such GFETs specifically immobilized with aptamers are shown to record even the tiniest concentrations of OTA in phosphate-buffered saline (PBS) solution, with a detection limit of 4 pg/mL. This new GFET aptasensor technology provides a rapid and accurate approach for the multiplex detection of different toxins in liquids for in-field use.

## 2. Results and Discussion

**Figure 2:**
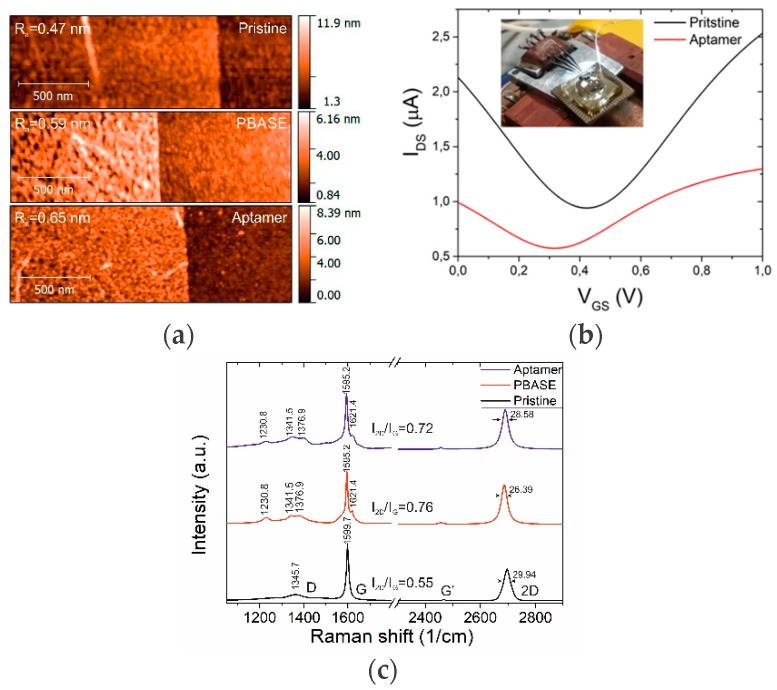
The characterization of a GFET aptasensor during assembly. (**a**) Change in the surface of the graphene channel during sensor layer assembly (inset: the values of surface roughness). (**b**) Charge neutrality point (CNP) shift for the GFET after aptamer linking (inset: photo of the GFET chip packaged in a PDMS chamber for liquid handling). (**c**) Raman spectra of pristine graphene (black), after exposure to the 1-pyrenebutyric acid N-hydroxysuccinimide ester (PBASE) solution (red), and after aptamer grafting (blue). Signature peaks of the noncovalent π−π interaction between PBASE and the graphene surface were observed after immersion in the PBASE solution.

The GFETs used in this work were based on single-layer CVD-grown graphene that is known to have excellent electrical properties and electrolyte stability [13]. To ensure electrical stability and the absence of signals originating not from graphene, the chip was passivated, leaving only the graphene area open to the electrolyte. A full fabrication overview is given in Section 3. We applied a bias voltage of 100 mV to the GFET and recorded the channel current, while the gate–source current was applied through an Ag/AgCl pellet electrode. Bare graphene (see Figure 1a) is typically p-doped, resulting in the charge neutrality point (CNP) being shifted to positive values, +200–300 mV typically. Every step of biomolecule functionalization, such as attachment of 1-pyrenebutyric acid N-hydroxysuccinimide ester (PBASE) and the OTA aptamer (see Section 3), resulted in the addition of extra free charges to the surface, which shifted the CNP position. Experimentally, we observed a gradual change in the optical and electrical properties accompanied by channel height increasing, showing the effect of the building blocks (PBASE and OTA aptamer) on the graphene characteristics (see Figure 2). The usage of PBASE as the covalent linker between graphene and an aptamer was recently introduced and is nowadays widely utilized [18]. PBASE linker provides long-term stability of sensing properties and reusability of the sensors. We performed atomic force microscopy studies of the graphene channel upon PBASE treatment to confirm the successful attachment of the molecules. As can be seen from Figure 2a, the deposition of molecular layers of PBASE and aptamer increased the roughness of the graphene surface from 0.47 nm to 0.59 nm (Figure 2a), which correlates with the height of formed layers [19]. Later addition of aptamer further increased the surface roughness up to 0.65 nm. Additionally, we confirmed the presence of aptamer electrically. The aptamer-functionalized GFETs measured with liquid gating demonstrated a prominent shift of the CNP from 0.42 V to 0.33 V, showing the electrostatic gating effect from an aptamer layer to the graphene channel (Figure 2b) [20]. Further characterization of graphene was carried out via Raman spectroscopy. The graphene-specific peaks were present in the spectrum after modification, demonstrating the stability of graphene towards applied chemistry (Figure 2c). Interestingly, we observed a set of new peaks in the range of 1230–1380 cm^−1^ and a single shoulder peak at 1621.4 cm^−1^ that corresponds to PBASE molecule attachment [15,20]. The modification of the graphene surface was additionally supported by the redshift of the G and 2D peaks and the increase of the 2D/G intensity ratio.

**Figure 3:**
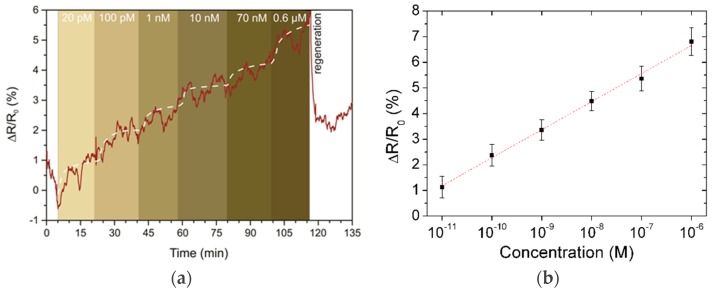
(**a**) GFET channel resistance changes upon sensing multiple concentrations of ochratoxin A (OTA) ranging from 20 pM to 0.6 μM. (**b**) A diagram of the relationship between saturated response (derived from the eye-line shown in white in (**a**)) and OTA concentrations.

Prior to the real-time toxin sensing experiments, a drop of 30 µL PBS solution with a pH of 7.4 was placed on the channel surface. To prevent solvent leakage, we covered the channel area with a PDMS ring. The GFET was biased to 0.1 V for current measurements. The addition of toxins was performed via stepwise drops (10 µL each) with concentrations ranging from 10 pM to 1 μM in tenfold steps. The final total concentrations are calculated later. We observed the stepwise growth of the graphene channel resistance during the toxin addition (Figure 3a). The non-monotonic resistance response to the OTA concentration can be explained by the saturation of aptamer sites on the graphene surface. The size of the graphene channel is 20 × 20 μm^2^, and the area of a PBASE molecule, which can bring only one aptamer, is ~0.052 nm^2^. Thus, we estimated the maximal amount of toxins absorbed onto the channel to be equal to 5.2 pg. As we gradually increased the concentration of OTA, adding 10 μL of the OTA solution each time, all active centers had to be occupied already once the level reached 10 nM. The observed slow growth in resistance we explain by diffusion of the toxin molecules in the vicinity of the graphene channel.

**Figure 4:**
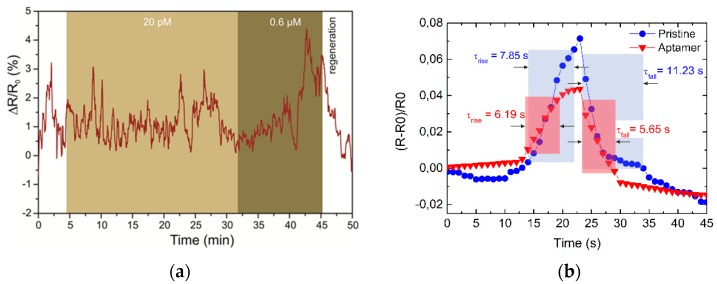
(**a**) The resistance response of pristine graphene channels to the presence of OTA. (**b**) The regeneration of aptamer-modified and nonmodified graphene channels. The red and blue squares show 10–90% thresholds of the resistance change during the regeneration procedure.

We performed the same characterization for five different GFET aptasensors, and the accumulated response of the sensors for a set of toxin concentrations is presented in Figure 3b. To demonstrate the specificity of the biosensors, i.e., the importance of aptamers, we performed the same set of experiments on a GFET with pure graphene channels (no PBASE, no aptamer functionalization, see Figure 4a). A noticeable response was observed only for the 0.6 μM concentration. This supports our observations of the aptasensor response at high OTA concentration when all the aptamer sites are occupied, and it can be explained by an increase in the intrinsic electrostatic gating effect from OTA molecules. From the data reported in Figure 3b, we estimate the detection range of the OTA aptasensors to be 10 pg/mL to 4 ng/mL. The detection limit (LOD) was evaluated from the noise characteristics of the GFET current for a signal-to-noise ratio of 3. The noise of the resistance change was calculated as 0.3% which corresponds to a LOD of 4 pg/mL. It should be noted that saturation of the sensor was weakly observed within the 20 min measurements for each step. Nevertheless, analysis of the resistance curve can provide an estimation of the binding process of about 5 min, demonstrating the high reaction speed compared to other on-chip techniques and making it appropriate for real-time monitoring of toxin concentrations.

The restoration of characteristics was done by washing the channel in 4 M urea solution in PBS. We calculated the rise and fall times for two devices using 10%–90% thresholds of the signal (Figure 4b). It is interesting to note that the restoration speeds for aptamer-modified and pristine graphene channels differed. The regeneration time of the OTA-specific aptasensor was about 5.6 s, while the GFET with pure graphene channels had a specific restoration time of 11.2 s. While the restoration of aptasensors depends on DNA molecular regeneration during denaturation in urea solution, which is very fast [21,22], physical cleaning of the surface can cause the regeneration of the pure graphene sensor during washing from absorbed molecules.

**Figure 5:**
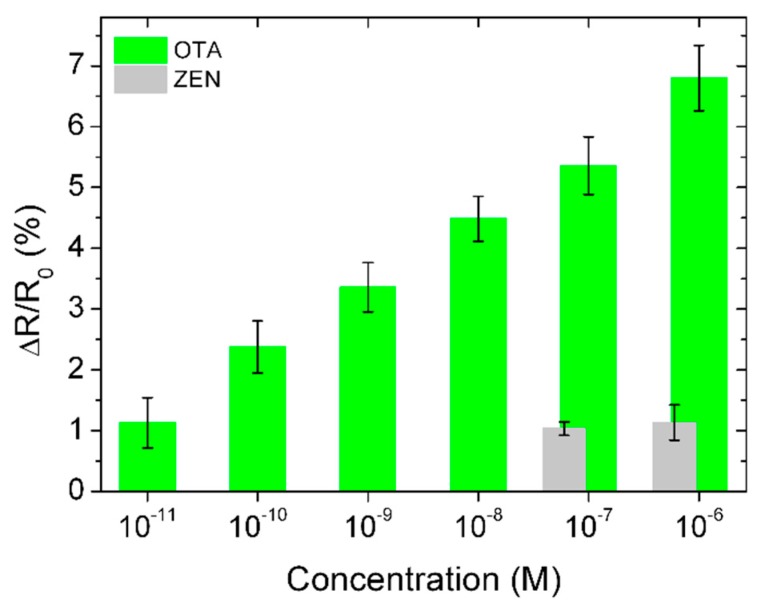
A signal response comparison of the developed GFET-based biosensors towards OTA (green, *n* = 5) and zearalenone (ZEN) (grey, *n* = 3) toxins, showing a clear specificity towards OTA.

To show the selectivity of the GFET-based aptasensor, we used it towards another toxin, zearalenone (ZEN). ZEN solution was dissolved in PBS in two concentrations, namely, 60 nM, and 600 nM. The sensor response to the 60 nM concentration was about 5 times lower than that to OTA (Figure 5). The response to 600 nM was about 7 times lower than that to OTA, and even smaller than that to 10 pM OTA, confirming the very high selectivity of the developed sensor. We should note that the response to ZEN of the OTA-aptamer-modified GFET slightly depends on the concentration, and it is on the same level as 10 pM for OTA, which limits the cross selectivity to these values.

To validate the developed aptasensors in real food samples, we prepared red wine spiked with OTA at 100 pM and 500 pM concentrations. As a low concentration of alcohol does not affect the binding process of OTA and the aptamer [23], we used 6k centrifuging to eliminate the massive particles from the sample prior to spiking. The sensitivity was measured in the same experimental conditions. Recovery of about 105% was observed for 100 pM and 120% for the 500 pM cases (see Table 1). The increases in recovery for higher concentrations can be explained by changes in the conditions of gating in the graphene channel in an acidic environment (wine). The response of the GFET aptasensors in different pH environments needs a more detailed study.

We should note that in the present study we used single transistors out of an array of 32 GFETs on a chip placed in an area of 1 × 1 mm. This structure can be prospectively used for multiplexed sensing [24] of different pathogens in food by proper microfluidics channel organization and functionalization, which is the aim of future research.

In conclusion, this work reports on the development of an ion-selective aptamer-modified GFET for real-time detection of ochratoxin A. A scalable and reproducible technological process of biosensor assembly integrated with conventional microelectronic technology was demonstrated. The fabricated aptasensor showed the capability to detect deficient concentrations of OTA down to 4 pg/mL in solution. A fast regeneration time by urea treatment makes this sensor an effective platform for low-cost bioanalytics. This work shows the general ability of graphene-based electronics for real-life toxin detection. We believe future technology will allow multiplexing of the recording signal on a single chip via specific surface modification for each probed toxin.

## 3. Materials and Methods

### 3.1. Graphene FET Fabrication

Single-layer graphene was CVD-grown on 25 µm thick copper foil for 30 min at 1050–1070 °C, and methane gas was used as a carbon source. The process was performed in a quartz tube furnace in the presence of an Ar/H_2_/CH_4_ gas mixture (300 sccm, 15 sccm, and 0.5–1.0 sccm, respectively). Further, a thin layer of poly(methyl methacrylate) (PMMA) was spin-coated on top of the graphene/copper stack and was used as a support layer during the transfer. In order to not waste a large fraction of the graphene, we used a modified, high-throughput transfer technique described elsewhere [25]. Prior to graphene transfer, the surface of a Si/SiO_2_ wafer was treated with oxygen plasma (0.8 mbar, 100 W, 5 min) in order to clean and increase the hydrophilicity of the surface, thereby improving the graphene-to-SiO_2_ adhesion. Then, we transferred PMMA/graphene stack onto the wafer and left it for 24 h under ambient conditions to dry out slowly. To re-flow the PMMA and improve the graphene-to-substrate adhesion, we annealed the wafer at 150 °C for 10 min [26]. Afterward, the PMMA was dissolved in acetone (one hour in 50 °C acetone followed by 12 h in cold acetone). Finally, the structure was washed with isopropanol (IPA) and DI water, dried under nitrogen flow, and annealed at 350 °C in N_2_ atmosphere. The graphene was then patterned via oxygen plasma etching (300 W, 200 sccm, 10 min). Using e-beam assisted evaporation and lift-off of LOR-3B and AZ nLOF-2020 photoresists, we deposited the 10 nm Ti and 100 nm Au metallization. Photostructurable polyimide (HD-8820, HD Microsystems) was spin-coated 3 µm thick as the last step of passivation to prevent current leakage during liquid measurements. After exposure (i-line, 250 mJ/cm^2^), development, and annealing with a slow ramp-up to 350 °C and slow cooling down, the polyimide layer formed a perfect, pinhole-free passivation covering the metal feedlines. The passivation covered all metallic feedlines as well as a partial area (<2 µm) of graphene–metal contacts, as shown in Figure 1a. The details of the fabrication process can also be found elsewhere [27]. Prior to the sensor assembly, the surface of the GFETs was UV treated in the air for 4 min to remove the organic residuals and activate the carbon bonds.

### 3.2. Aptamer Covalent Bonding

An HPLC-purified OTA aptamer with a sequence of GAT CGG GTG TGG GTG GCG TAA AGG GAG CAT CGG ACA [28,29,30] and an amino-modified 5′ end was purchased from Metabion AG (Germany). It was dissolved in freshly prepared PBS buffer with pH 7.4. To activate the graphene surface, the GFET chips were soaked in 1 mM PBASE solution in dimethylformamide (DMF) for 6 h. The chips were then washed with DMF, IPA, and DI water consecutively, for 3 min each. Finally, the chips were dried with an airgun. OTA aptamer dissolved in PBS (5 μM) was covalently bonded to the PBASE by an *N*-hydroxysuccinimide cross-linking reaction [31]. The process was carried out for 4 h in a humid atmosphere to prevent solvent evaporation. The unreacted aptamer was washed in a PBS bath (2 min) and dried using an airgun. Finally, surface passivation was performed in 100 mM ethanolamine (in PBS) for one hour to deactivate the nonlinked reactive groups remaining on the graphene surface.

### 3.3. Ochratoxin A and Zearalenone Solution Preparation

OTA solution in acetonitrile (10 μg/mL) and ZEN solution in acetonitrile (50 μg/mL) were purchased from Sigma-Aldrich (Laramie, WY, USA). They were titrated in PBS solution several times to achieve the required concentration. Each concentration in a volume of 10 μL was added to the sensor surface during the measurements.

### 3.4. Recovery Study

Dry red wine (14% Cabernet Sauvignon/Merlot, France, 4 years aging) was bought in a local store. The wine samples were spiked with a known concentration of OTA solution in PBS to receive final concentrations of 100 pM and 500 pM.

### 3.5. Graphene FET Characterization

At all stages of graphene channel modification, we measured the electrical characteristics via a semiconductor analyzer (MNIPI, Minsk, Belarus). Raman spectra were recorded on a Centaur HR Raman spectrometer (Nanoscan Technology, Dolgoprudnyy, Russia) with a 100× objective at 532 nm (Cobolt, Solna, Sweden) with a beam spot of ~1 μm^2^ and laser power of 0.5 mW. A Solver Pro atomic force microscope (NT-MDT, Moscow, Russia) was used to study the morphology of pristine graphene and after its modification with PBASE and aptamer. All the experiments with toxin detection were carried out in lab air. During these measurements, GFETs were connected to an IPS-16 gas sensor meter (Ecological sensors and systems, Moscow, Russia) to collect the resistance signal.

## Figures and Tables

**Table 1:** Application of the aptasensor for the determination of OTA in wine samples.

Samples	Spiked, pM	Detected, pM	Recovery, %
1	100	105	105
2	500	600	120
